# Lactic acid production by *Enteroccocus faecium* in liquefied sago starch

**DOI:** 10.1186/2191-0855-2-53

**Published:** 2012-09-28

**Authors:** Cirilo Nolasco-Hipolito, Octavio Carvajal Zarrabal, Rubena Malfia Kamaldin, Ling Teck-Yee, Samuel Lihan, Kopli Bin Bujang, Youji Nitta

**Affiliations:** 1 Faculty of Resource Science and Technology, Universiti Malaysia Sarawak, 94300, Kota Samarahan, Sarawak, Malaysia; 2 Biochemical and Nutrition Chemistry Area, University of Veracruz, SS Juan Pablo II s/n, Boca del Río, CP 94294, Veracruz, Mexico; 3 SpeCorp, Sdn Bhd, Level 2 Block 218 KNLD., Jln Tun Ahmad Zaidi Adruce, Sarawak, Kuching, 93200, Malaysia; 4 The College of Agriculture, Ibaraki University, 3-21-1, Chuuo, Ami, Inashiki, Ibaraki, 300-0393, Japan

**Keywords:** *Enteroccus faecium*, Lactic acid, Repeated batch fermentation, Liquefied sago starch, Cell reuse

## Abstract

*Enterococcus faecium* No. 78 (PNCM-BIOTECH 10375) isolated from *puto,* a type of fermented rice in the Philippines was used to produce lactic acid in repeated batch fermentation mode. Enzymatically liquefied sago starch was used as the sole carbon source, since sago (*Metroxylon spp)* is a sustainable crop for industrial exploitation. Liquefied sago starch was inoculated with *E. faecium* to perform the saccharification and fermentation processes simultaneously. Results demonstrated that *E. faecium* was reused for 11 fermentation cycles with an average lactic acid yield of 36.3 ± 4.71 g/l. The lactic acid production was superior to that of simple batch mode and continuous fermentation in terms of lactic acid concentration. An un-dissociated lactic acid concentration of 1.15 mM affected the productivity of the cells. Work is in progress to maintain and increase the usability of the cells over higher fermentation cycles.

## Introduction

Lactic acid is an important commodity because it is a multi-functional versatile organic acid having a wide range of applications. One of the most important factors that affect the overall production cost in Lactic Acid Fermentation (LAF) is the raw material. The consortium CSM ( [2010]) reported that the raw material costs for lactic acid (LA) production, as a percentage of sales, increased from 52.9% in 2009 to 53.2% in 2010. For a long time, it has been stated that lignocellulosic materials are very promising for bio-refinery applications, but this technology is still problematic (Zhou et al. [2011]). Recently Ou et al. ( [2011]), reported a process using *Bacillus coagullans* to produce LA from non food carbohydrates, and interestingly, an indigenous *Clostridium phytofermentans* was found as a potential microorganism for the efficient use of lignocellulosic materials to produce ethanol, hydrogen and organic acids ( [Leschine and Warnick 2010])*.* Among starchy materials, sago starch is being considered as an attractive raw material for food and industrial exploitation due to the fact that it is produced abundantly in the agricultural plant *Metroxylon spp* (Karim et al. [2008]). In 2008, Malaysia exported 37,365.3 metric tons of sago flour, thereby earning RM44, 091.0 million (Malaysia Dept [Statistics 2011]). The sago palm efficiently fixes carbon dioxide to synthesize starch in large quantities in its trunk. Sago starch granules are generally bigger than those of rice, (3–10 μm), corn (5–20 μm), wheat (22–36 μm), or cassava (5–25 μm), but smaller than those of potato (15–85 μm) (Nor- [Nadiha 2010]). Sago starch contains approximately 74-80% of amylopectine and 24-31% of amylose (Karim et al. [2008]) and has a crystalline structure (Yetti et al. [2007]). These properties of sago starch make difficult its hydrolysis. Uthumporn et al. ( [2010]) reported that the relative order in the hydrolysis of the starchy materials studied was as follows: corn starch > mung bean starch > cassava starch > sago starch. These findings demonstrated that sago is a difficult substrate for raw starch degrading enzymes (Yetti et al. [2007]). On the other hand, an improvement in the industrial production and efficiency of enzymes has decreased their cost in the market (Novozymes and BBI [International 2005]). Nevertheless, to improve the economics of LAF, the use of microorganisms with amylolytic activity could be preferred because it saves in terms of enzymes and energy in the liquefaction/saccharification process. Some strains of fungi and bacteria capable of producing LA directly from starchy materials by using different strategies have been reported in the literature (Lu et al. [2009]; [Petrova and Petrov 2012]; Shibata et al. [2007]; Xiao et al. [2011]). For instance, the amylolytic bacterium *L. amylovorus* NRRL B4542 is reported to be capable of fully converting liquefied corn starch to LA, with a productivity of 25 g/lh in continuous culture through the use of a yeast extract concentration as high as 30 g/l as nitrogen source ( [Zhang and Cheryan 1994]). On the other hand, Shibata et al. ( [2007]) reported the use of *Enteroccus faecium* 78 as a promising microorganism to produce L-(+)-LA directly from raw sago starch (RSS) in continuous culture by using a hollow fibre cartridge to recycle the cells. It was reported that *E. faecium* 78 performed well at 30°C and pH 6.5 with a productivity of 3.04 g/lh at a LA concentration as low as 16.6 g/l (Shibata et al. [2007]). In their research the fermentation mode was of capital importance to enhance the productivity of the system. In this regard the repeated batch fermentation (RBF) process combines the advantage of batch and fed-batch fermentation processes mainly making possible to conduct the process by long periods and improving the productivity compared to batch process (Treichel et al. [2010]). RBF reduces the cost of fermentation process and enhance the productivity through the use of high cell density (Yamakawa et al. [2010]). Moreover, from an industrial point of view by using the RBF mode the production period can be shortened, compared to standard fed-batch or batch processes resulting in a significant increase of the final product yield (Russ et al. [2007]). Therefore, in this study we used the RBF for LA production with the strain *E. faecium* 78 (PNCM-BIOTECH 10375) in liquefied sago starch (LSS) as the only carbon source. In general, the main objective was to improve the productivity of the system, which includes the use of LSS, recycling of the yeast to speed-up the fermentation process.

## Materials and methods

### Sago starch hydrolysis

Industrial grade sago starch was obtained from Nitsei Sago Industries, Kampung Teh, Mukah, Sarawak. The hydrolysis of sago starch has been reported elsewhere (Carvajal et al. [2009]). Briefly and just for the LSS, 400 g of sago starch (dry basis) were suspended in tap water and the final volume was adjusted to 1 litre. The pH of the suspension was adjusted to 4.5. A thermostable α-amylase (1,4-α-D-glucan glucanohydrolase) (EC 3.2.1.1). from *Bacillus licheniformis*, 240 KNU-S/g of starch, (10 μl, 3000 U/ml) (Novozyme Co.) was added to liquefy the starch at 95-100°C for 2 hours. This LSS was cooled and further treated with 0.5 μl (0.23 amyloglucosidase units AGU) of enzyme dextrozyme DX (Novozyme, Co) at pH 6.5, heated at 60°C, for 24 h and agitated at 300 rpm to produce the hydrolysed sago starch (HSS). The gelatinised sago starch (GSS) was prepared by heating at 65°C a slurry of raw sago starch at concentration of 20 g/l and agitated at 300 rpm. The raw sago starch (RSS) used as substrate was prepared just by suspending 20 g of sago starch in 1 l of tap water. Previously the sago starch was dried at 105°C for 1 hour.

## Culture conditions

### Microorganism

*Enterococcus faecium* No. 78 (PNCM-BIOTECH 10375) was used throughout this study. Stock cultures were maintained in PDA media at −84°C. One vial containing the frozen strain was thawed and used to inoculate 10 ml broth containing 20 g/l LSS and 5 g/l of yeast extract (YE), and the broth was incubated for 24 h at 36°C in static conditions.

### Culture media

The media for LA production was prepared with LSS and supplemented with YE at a concentration of 5 g/l (Difco, USA). The YE was dissolved in 100 ml of water and sterilized separately at 121°C, for 15 min and added to the hot LSS sterilized at 121°C for 15 min.

### Dry cell weight

The dry cell weight (DCW) was determined by a pre-established calibration curve of optical density (OD) against DCW. To determine DCW, samples of 200 ml of fermented broth at different OD were centrifuged at 10000 rpm for 10 min. The harvested cells were washed with 0.2 M hydrochloride acid and centrifuged again under the same conditions. This procedure was applied three times. The harvested cells were dried at 60°C for three days, then the weight was registered and the OD plotted against the DCW. It was determined that one OD unit was equivalent to 6.4 g/l DCW.

## Fermentation mode

### Batch fermentation

The fermentations were carried out in a 3 litre Jar fermenter fully controlled by a computer system. Parameters such as temperature, pH, agitation, cell concentration, and sodium hydroxide consumption were monitored on-line in real time. The OD was monitored by a laser sensor, model LA 300-LT (Automatic Research System, Tokyo, Japan) at 780 nm. The temperature was controlled at 30°C and the pH was controlled at 6.5 (Shibata et al. [2007]). The agitation was controlled at 200 rpm. The media used contained LSS at 20 g/l and 5 g/l of YE. The pH was controlled with 10 M sodium hydroxide.

### Repeated batch fermentation

The medium containing LSS at a level of 20 g/l was maintained until the fourth fermentation cycle. Then, it was increased to 40, 60, 80, and 100 g/l for the subsequent fermentation cycles. The operating conditions were: temperature 30°C, agitation 150 rpm, and pH controlled at 6.5 by direct titration with 10 M NaOH. The YE was maintained at a level of 5 g/l in all the fermentation cycles. The cells were harvested by centrifugation at 8,000 rpm for 10 min. The process of harvesting and re-suspending the cells was repeated for the subsequent cycles to perform the RBF mode.

### Analysis

Glucose and lactate were determined by an HPLC (LC-10 AD, RID-6A Refractive Index Detector, Shimadzu, Kyoto, Japan) using an Aminex HPX-87 H column (Biorad, CA., USA) at 50°C, with 0.6 ml/min flow rate of 5 mM H_2_SO_4_ as a mobile phase. Total organic acid was measured by direct titration in the fermenter through the addition of 10 M NaOH with a density of 1.3 g/l. The weight of NaOH used to neutralize the LA was recorded on-line in real time by a software in a computer and reported as LA by using the factor 0.69.

### Scanning electron microscopy

Air-dried sago powder was scattered on the specimen stub, then coated with OsO_4_ and platinum to strengthen the contrast for observation. The specimens were observed with a scanning electron microscope (JSM6360A, JEOL, Tokyo).

## Statistical analysis

Data from two replicate experiments and three repetitions of the analysis for each sample were expressed as the mean ± standard deviation (± SD). All analyses were conducted with PASW 18.0 software.

## Results

### Effect of sago starch in different forms on biomass production

**Figure 1:**
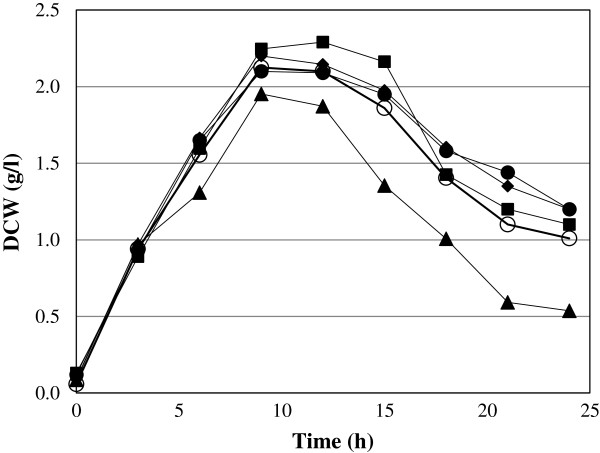
**Kinetics of biomass production by *E. faecium *using different forms of sago starch.** Symbols: (♦) Glucose, () Raw sago starch, (▴) Gelatinized sago starch, (○) Hydrolysed sago starch, and (□) Liquefied Sago starch. Fermentation were carried out in 3 L Jar fermenter, with 2 L working volume, pH 6.5, agitation 200 rpm and temperature of 30°C.

**Figure 2:**
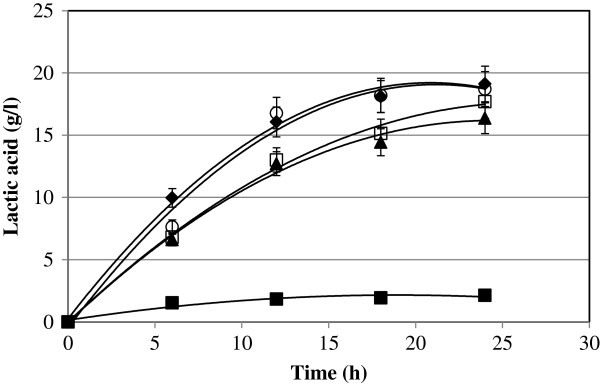
**Kinetics of lactic acid production by *E. faecium using *different forms of sago starch.** Symbols: (♦) Glucose, () Raw sago starch, (▴) Gelatinized sago starch, (○) Hydrolysed sago starch, and (□) Liquefied Sago starch. Fermentation were carried out in 3 L Jar fermenter, with 2 L working volume, pH 6.5, agitation 200 rpm and temperature of 30°C.

This experiment had the objective to justify the use of liquefied sago starch as carbon source for lactic acid fermentation. Sago starch as substrate in different forms was compared with glucose to study its effect on the growth of *E. faecium* and showed in the Figure 1. The results obtained showed that the RSS produced the lower biomass concentration compared with LSS, GSS, HSS and with glucose. The maximum biomass produced was only 1.95 g/l of cells and was significantly different compared with glucose, GSS, HSS and LSS, which produced a concentration of cells (2.1, 2.15, 2.25 and 2.12 g/l respectively). It was observed that RSS resulted in faster and shorter decaying in the biomass production. Regarding the production of lactic acid the strain was able to use all the forms of sago starch. However, the less suitable starch form was the raw sago starch, as it was expected. The maximum lactic acid concentration using glucose as carbon source was 19.1 g/l and for GSS, HSS, and LSS was 16.4, 18.7, and 17.7 g/l respectively. It was evident that RSS is a difficult substrate for *E. faecium* since it produced lower cell concentration and consequently very low lactic acid production. There was significant difference between RSS and the others sago starch forms (p < 0.05). The trend obtained in the preference for the substrate were GLU > HSS > LSS > GSS > RSS. With these results it was assumed that glucose cannot be used as substrate because it is expensive. HSS needs the whole liquefaction/saccharificaion process therefore energy and more enzymes for the saccharification is required. Gelatinized sago starch could be an option; however, it is complicated to get the gelatinization point in bigger scale. As can be observed in the Figure 2, raw sago starch produced lower concentration of lactic acid, using only 20 g/l of starch, which is very low for industrial purposes. Therefore, it was considered that liquefied sago starch can be the substrate for lactic acid fermentation and it was tested in repeated batch fermentation.

## Repeated batch fermentation

### Effect on biomass production

**Figure 3:**
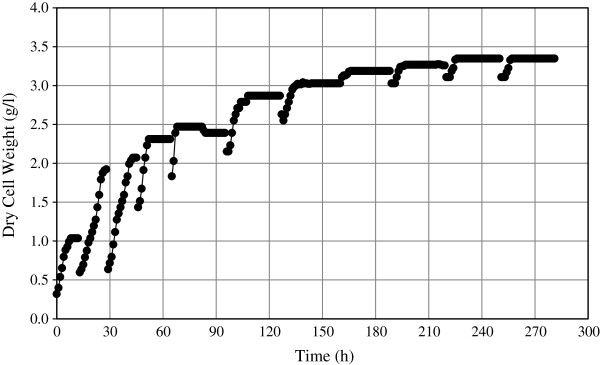
**Biomass production by *E. faecium *cultivated in liquefied sago starch in repeated batch fermentation mode during eleven fermentation cycles.** Fermentation were carried out in 3 L Jar fermenter, with 2 L working volume, pH 6.5, agitation 200 rpm and temperature of 30°C.

**Figure 4:**
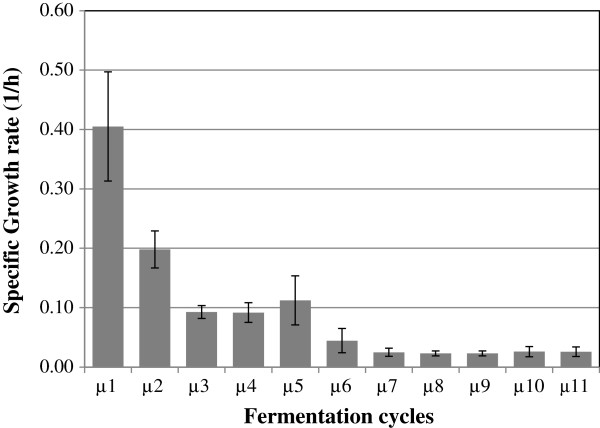
**Maximum growth rate (μ) of *E. faecium *during the log phase in the eleven fermentation cycles of both experiment of repeated batch fermentation.**

Figure 3 shows the biomass production during the RBF process. During the first four fermentation cycles, the LSS concentration was only 20 g/l to promote the growth of the cells. The production of biomass was approximately 1.04, 1.79, 2.07, and 2.31 g/l for the cycles 1, 2, 3, and 4 respectively in 12 h fermentation. In the sixth cycle the cells maintained a longer growing phase and concordantly this cycle produced higher LA concentration (42.5 g/l). In the cycles 9, 10 and 11, the growth was only for a period of almost 4 hours, and subsequently the cells started to decrease in their activity. Figure 4 shows the maximum specific growth rate (μ) during the eleven fermentation cycles as the average of both RBF fermentations reported in this study. *E. faecium* was able to maintain a growth rate of between 0.1-0.4 h^-1^, at substrate concentration of 20 g/l. Higher concentration of LSS decreased the growth rate at values lower than 0.05 h^-1^. From the cycle number six to eleven, the growth was only 0.028 ± 0.006 h^-1^.

### Lactic acid production in repeated batch fermentation

**Figure 5:**
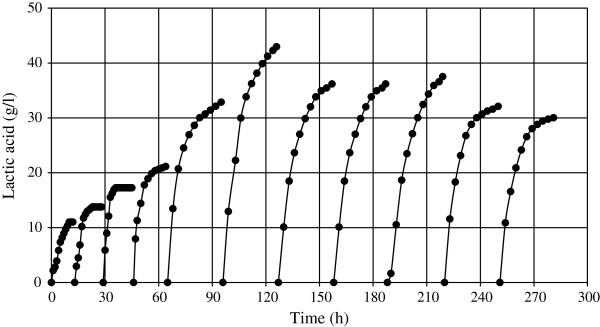
**Kinetics of lactic acid production by *E. faecium *cultivated in liquefied sago starch in repeated batch fermentation during eleven cycles.**

Figure 5 shows the production of LA in RBF during the eleven fermentation cycles. As explained above, the first four fermentation cycles were performed to increase the biomass concentration and because of this, a low concentration of starch was used. Thereafter, the starch concentration was increased and, as a result, the productivity of the system improved concordantly.

Table 1 shows the performance of *E. faecium* from cycles 1 to 5 and from 6 to 11 in 15 h of fermentation as the mean of the parameters reported. During the cycles 1 to 5 a volumetric productivity of 1.28 g/lh was obtained, very similar to that obtained by [Petrova and Petrov (2012]) using the strain *Lactobacillus paracasei* B41. From the cycles 6 to 11, the volumetric productivity increased as a consequence of higher biomass concentration and higher starch concentration. Higher LA improved the specific productivity from 9.86 to 11.45 g_LA_/g_DCW_, since biomass was increased from 1.95 ± 0.54 to 3.17 ± 1.86 and LA increased from 19.2 ± 8.5 to 36.3 ± 4.71 g/l. 

**Table 1:** **Overall productivity of the repeated batch fermentation process in two stages during eleven cycles for the production of lactic acid in liquefied sago starch by *E. faecium***

**Parameter**^a^	**Cycles 1-5**	**Cycle (6–11 )**
LA concentration (g/l)	19.2 ± 8.5	36.3 ±4.71
Volumetric productivity (g/lh)	1.28 ± 0.24	1.96 ±0.24
Specific Productivity ( gLAgDCW)	9.86 ± 0.35	11.45 ± 1.13
Yield_LA/TS_ (g/g)	0.47 ± 0.021	0.57 ± 0.14
Residual glucose	4.8 ± 4.01	3.17 ± 1.86
Dry Cell weight (g/l)	1.95 ± 0.54	3.17 ± 0.19

^a^Data were obtained as the mean of the values of each fermentation cycle where maximum productivity was observed.

**Figure 6:**
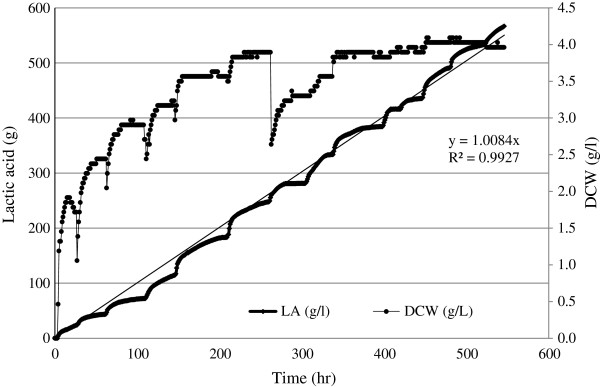
**Lactic acid and biomass kinetics by *E. faecium *cultivated in liquefied sago starch in repeated batch fermentation.**

In order to investigate whether a longer fermentation time in each cycle could increase the concentration of lactic acid, a longer RBF was performed (Figure 6). The table 2 summarized the overall productivity of the eleven fermentation cycles of this fermentation. The cells were able to maintain a productivity of 1 g/lh during 566 h of fermentation in RBF mode. Concordantly with this volumetric productivity and fermentation time, 1136.7 g of LA was produced in the whole process as recorded by the direct titration of the LA by 10 M NaOH. This quantity of LA produced in the whole fermentation from 1320 g of starch (dry basis) is equivalent to a conversion of starch to LA of 86.1%.

**Table 2:** **Overall productivity of the repeated batch fermentation after eleven cycles for the production of lactic acid in liquefied sago starch by *E. faecium ***

**Parameter**	**Value**
Working volume (l)	2
Total fermentation time (h)	566
Total Starch used dry basis(g)	1320.0
Total NaOH used in the titration of LA (g)	505.2
Total Lactic acid produced (g)	1136.7
Final Dry Cell weight (g/l)	3.9
Volumetric productivity (g/lh)	1.004
Yield ( gLAgstarch)	0.861

Figure 6 shows that *E. faecium* can keep the trend of LA production and that longer fermentation time did not improve the productivity of LA in terms of volumetric productivity. The cells were growing in each subsequent fermentation cycle until the sixth cycle and then the trend was asymptotic. At the end of the sixth cycle, the cells (3.9 g/l) were recovered and almost 33% of the pellet was not recycled with the aim to stimulate the cell growth having more availability of nitrogen source. Therefore, a decrease in the cell population was observed at the beginning of the seventh cycle up to 2.6 g/l. However, *E. faecium* very soon recovered its ability to grow again, and in a shorter time the same concentration as in the sixth cycle was reached again by the eighth cycle. Thereafter, the cells maintained the same level of low but active growth to produce LA. In this fermentation and in the previous one, the substrate consumption is indirectly measured as the production of lactic acid. The releasing of fermentable sugars, in this case, mainly glucose, immediately was consumed by the microorganism. The substrate was not the same since it was continuously changing due to the enzymatic hydrolysis performed by the amyloglucosidase in the fermentation media. Therefore, the glucose consumption rate was a function of the lactic production rate.

## Discussion

It was thought that glucose and hydrolysed sago starch (HSS) could promote the growth of the cells better than raw sago starch (RSS) and gelatinized sago starch (GSS). However, the trends obtained in all cases were similar, although it was observed that in the log phase the uptake of the GSS and RSS forms was slightly faster than that of the glucose and HSS. Similar observation was reported by Shibata et al. ( [2007]). They found that the performance of *E. faecium* using RSS was better than with corn, potato and wheat starches. It could be desirable to have RSS as the most appropriate form of starch to be used as direct substrate for the fermentation process; however, it is problematic to manage the RSS slurry at high concentration. Although Shibata et al. ( [2007]) did not reported how they prepared the raw sago starch, and if they sterilized the sago slurry, then; the form of starch used perhaps was the gelatinised sago starch. The gelatinised sago starch can be used as substrate but a very low concentration because when the concentration of starch is increased the viscosity of the media is very high. The viscosity of the slurry increases proportionally with the concentration of the starch. In general, when the starch concentration increased, sedimentation occurred due to saturation or insolubility. This situation was visualized by [Zhang and Cheryan (1994]); therefore, these researchers used liquefied starch to avoid these problems and they improved the process by using the amylolytic *Lactobacillus amylovorus* strain*.* Shibata et al. used *E. faecium* No. 78 cultivated in RSS at a concentration no higher than 20 g/l, which is very low for an industrial application. Therefore, it could be reasoned that using batch and its extension RBF might be advantageous to produce higher LA concentration at a similar production rate.

The RBF mode was applied with the aim to reuse the cells in order to improve the overall productivity of the fermentation. One of the key points in fermentation technology is to maintain the cells in a stable and perpetual state of productivity and to avoid damage to the cells, the aim being to increase their reusability for longer periods. This pattern is well observed in ethanol fermentation where the yeast can be reused 400–600 times, but only if the concentration of ethanol is 7-10%. As reported by Amorim et al. ( [2011]) higher ethanol concentration affects the viability of the cells and their reusability falls notably. Similarly, longer exposition of *E faecium* to a high concentration of LA resulted in lowered productivity. This fact was observed as the behaviour of the specific growth rate, which was affected by the LA. In Figure 4 it was observed that the maximum specific growth (μ_max_) was found at a low concentration of starch and higher availability of a nitrogen source; for instance, in the first cycle to synthesize the necessary proteins for the metabolism of *E. faecium*; especially, the synthesis of amylases to produce glucose for growth was observed as biomass production. In addition, due to the low concentration of glucose the effect of osmotic stress was avoided, which could help in the productivity of the cells. In subsequent cycles, the growth rate decreased, and contrary to the first cycle, less availability of nitrogen source for a higher population of microorganisms decelerated the growth. Although the nitrogen source could affect the growing of the cells the general trend was a slow growing rate and maintained LA productivity.

On the other hand, as other studies have found, there is an optimum concentration of starch around 20–60 g/l, where the fermentation can go faster but is not limited by this concentration (Narita et al. [2004]; Naveena et al. [2005]; Okano et al. [2009]; [Petrova and Petrov 2012]; Yun et al. [2004]). It is true that a higher concentration of starch to produce higher LA titter is possible, but the time process will also increase proportionately. To produce the highest concentration of 42.5 g/l of LA using *E faecium* and LSS it took 30 h, approximately (Figure 5). Using raw starch, ( [Petrova and Petrov 2012]) reported the highest amount of LA (37.3 g/l) from 40 g/l of raw starch during 48 h of fermentation using *Lactobacillus paracasei* B41. In our study it was observed that this concentration of LA has a strong effect on the growth of *E. faecium,* because after this concentration the production of LA was very limited. After the said concentration, the productivity decreased and the fermentations stopped with an asymptotic trend. Although using LSS form had some advantages because the effect of osmotic stress that could impair the cells as a result of glucose being used directly was avoided, the productivity production of LA was only around 36.3 g/l (Table 1). The fact that *E. faecium* has a faster glucose uptake compared with other microorganisms, especially those having amylolytic abilities such *L amylovorus*, *L. manihotivorans* (Shibata et al. [2007]), could be valid only at a low starch concentration (less than 20 g/l).

On the other hand, if it is possible to increase the biomass by increasing the nitrogen source to improve the productivity; then it is possible to reduce the fermentation time and increase the LSS concentration. Here, the key point is to decrease the exposure time of the cells to LA. This same situation applies also for ethanol fermentation (Amorim et al. [2011]); when ethanol concentration increases, the yeast starts to lose activity due to a high percentage of the active biomass dying. Then, the numbers of cycles to reuse the cells becomes reduced and the overall productivity of the system fails. Shibata et al. ( [2007]). only used a concentration of no more than 20 g/l and the dilution rate was low, because increasing the dilution rate is probably a way to wash out the amylolytic enzymes produced by *E. faecium*, although there are reports that amylase enzymes could bind to the cell membrane ( [Walker 1965]; Anderson et al. [1989]). Then, the system becomes inefficient at a high dilution rate and a high concentration of sago starch and, even worse, because of the clogging of the membrane system at a high concentration of suspended solids ( [Zhang and Cheryan 1994]).

**Figure 7:**
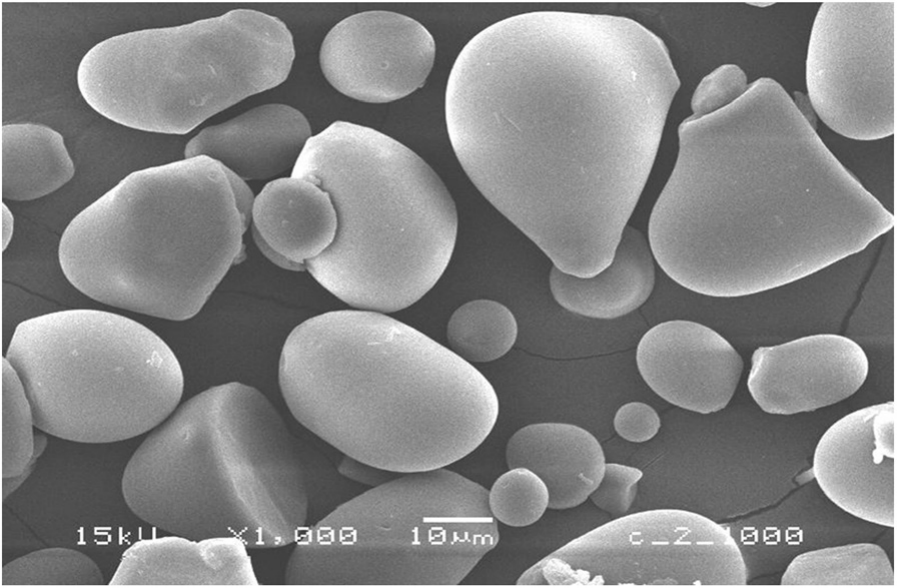
**SEM photograph of starch granules from Sarawak.** Bar: 10 μm.

Moreover, it was reported that sago starch was a difficult substrate for the amylolytic enzymes, due to the lack of suitable surface ready for the attack of the enzymes, and that it did not have natural pores or deep channels to facilitate the action of the enzymes (Uthumporn et al. [2010]; Nor Nadiha et al. [2010]). It was observed through SEM that sago starch has a very smooth surface and granule size of 6–50 μm (Figure 7), having a distribution of 16% of particles with a size of 42 μm, which agrees fairly well with the finding of Nor Nadiha et al. ( [2010]) and Nitta et al. ( [2008]). 

The characteristics of sago starch can be overcome with the use of Termamyl® SC to liquefy the sago starch with the aim of facilitating the subsequent attack of the amylase produced by *E. faecium*. The enzyme termamyl can only produce oligosaccharides and not glucose. In doing so and taking into account that the liquefaction process takes less than 2 h, and that the actual cost of Termamyl® SC is reasonably cheap, it can be used for this purpose.

For instance, the cost of Termamyl is around US$ 6715.60/ton and the dose of enzymes per ton of starch is around 0.625 kg, which means $4.20 US/ton of starch (Alagaratnum 2011, personal communication). Through a simple calculation and taking into account the price forecasted by Energetics [Incorporated (2003]) of about 550.0-1100.0 $US/ton, and an efficiency of 90% of conversion from starch to LA, as the percentage of the enzyme cost in the total cost of LA production could be considered as low (0.60%). Yet, this percentage in the total cost could be even lower if we consider the higher price for the LA food grade produced by Purac.

On the other hand it was observed that for this specific strain of *E. faecium* and from these results obtained, it was assumed that the LA concentration which induced the growth inhibition of *E. faecium* was in the range of 45 g/l (500 mM). Assuming that this is the growth inhibition concentration, with the LA pKa of 3.86, and using the following equations:

(1) $$pKa=pH-Log\left(\frac{L^{-}}{HL}\right)$$

(2) $$\left[L^{-}\right]+\left[HL\right]=500\text{mM}$$

where [L-] is the dissociated and HL the non-dissociated LA, the fermentation was controlled at pH 6.5, and the concentration of non-dissociated LA that produced the growth inhibition of *E. faecium* was calculated at approximately 1.15 mM. This critical concentration of LA which affects the growth of *E. faecium* was lower compared to those reported for *Lactobacillus rhamnosus* (4.7 mM) and *Lactobacillus helveticus* (5 mM) (Gonçalves et al. [1997]; [Timmer and Kromkamp 1994]). Therefore, it is suggested that *E. faecium* lowered the rate of LA production when it starts to reach a concentration close to 45 g/l and then modified its metabolism to synthesize exo-polysaccharide. *E. faecium* was able to grow at a rate as high as 0.4 h^-1^ in the first fermentation cycle. In the second cycle μ decreased to 0.27, then to 0.11, and from the fourth to eleventh cycle μ was almost constant at level of 0.028-0-0.02 h^-1^ (Figure 4). Even at low growth rate the strain maintained its LA production rate at a level of 1 g/lh.

## Conclusion

In conclusion, *Enterococcus faecium* No. 78 (PNCM-BIOTECH 10375) isolated from *puto,* a type of fermented rice in the Philippines, was able to produce LA in RBF mode. The cells retained their metabolic activity during 566 h of fermentation. However, the growth of this strain was affected strongly by non-dissociated LA at a concentration of around 1.15 mM, by lowering the productivity of the system.

## Competing interests

The authors of this manuscript declare that do not have any financial or non-financial competing interest in the submission of this experimental results in the journal AMB EXPRESS.

## Authors’ contributions

The authors declare that we belong to a group of researcher with common interest on research. The next are our individual contribution for the manuscript ID 2135787809763144 titled Lactic acid production by *Enteroccocus faecium* in liquified sago starch. CNH: The performed fermentations in repeated batch fermentation mode and drafted the manuscript. OCZ: The preparation of the manuscript and worked in the hydrolysis of the sago starch as well drafted the manuscript. RMK: The fermentations testing all the forms of sago starch and did the analysis needed during the research. LTY: The statistical analysis with its corresponding interpretation of the results. KB: Contributed with the preparation of the manuscript. SL: Contribute with the redaction of manuscript. YN: The scanning electron microscopy pictures for the starch granules. All authors read and approved the final manuscript.
